# Supplementary figures and images for: Single-cell RNA-sequencing reveals pre-meiotic X-chromosome dosage compensation in Drosophila testis
Source: PLoS Genet. 2021 Aug 17;17(8):e1009728. doi: 10.1371/journal.pgen.1009728 (PMC8396764; doi:10.1371/journal.pgen.1009728)

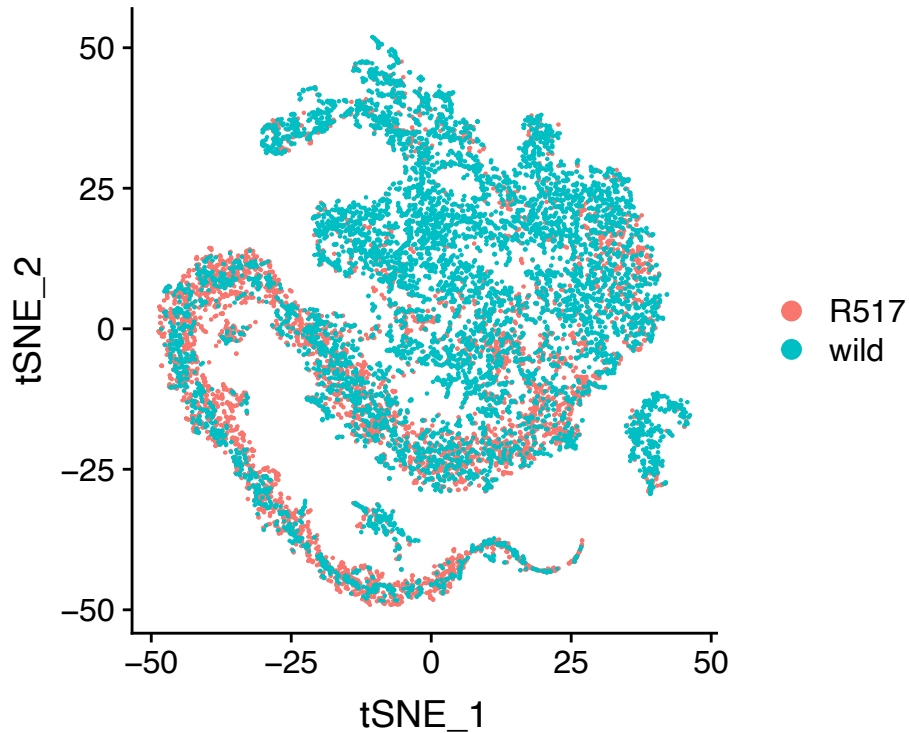

Supplement: S1 Fig — Both strains overlap well. Cell types were assigned from this integrated dataset. (PDF) [file pgen.1009728.s001.pdf]

Identity

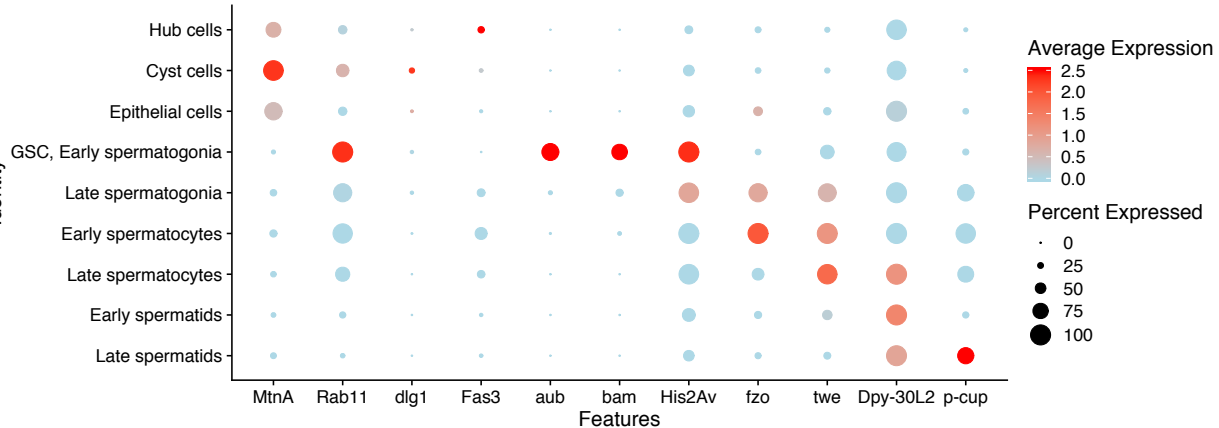

Supplement: S2 Fig — These genes were used to assign cell types, with details in the methods section. (PDF) [file pgen.1009728.s002.pdf]

Cell type

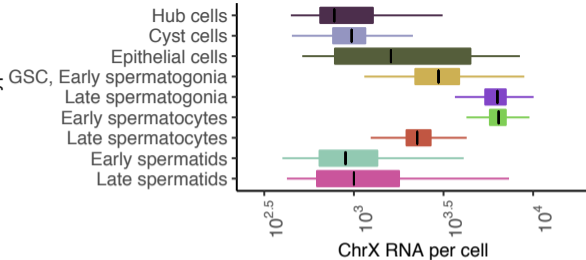

Cell type

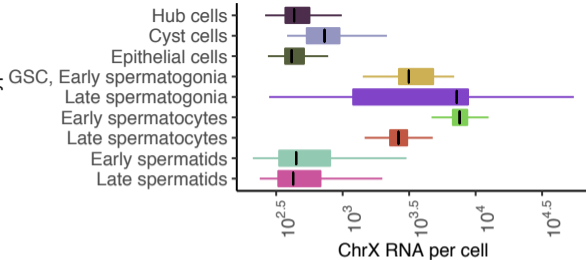

Cell type

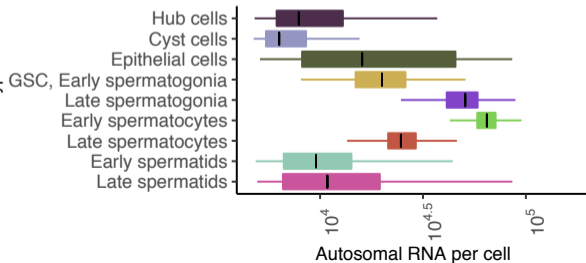

Cell type

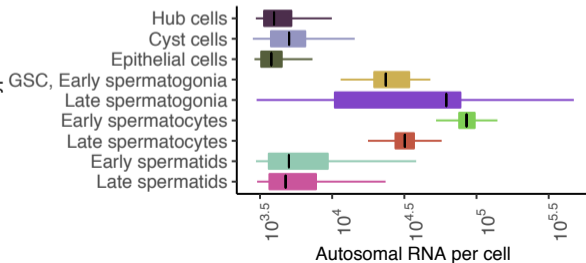

Supplement: S4 Fig — Left is R517, right is wild type. This corresponds to Fig 1C and 1D. (PDF) [file pgen.1009728.s004.pdf]

Cell type (R517)

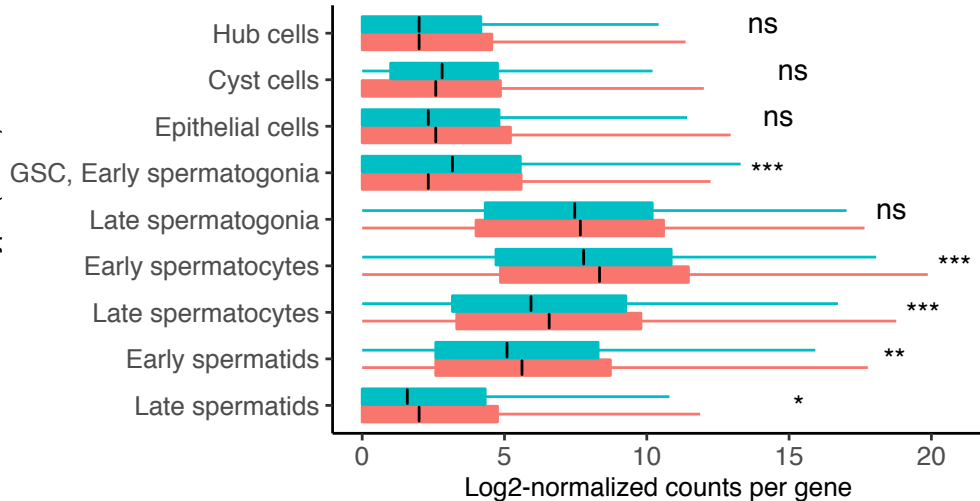

Chromosome

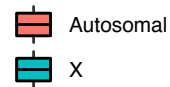

Cell type (Wild)

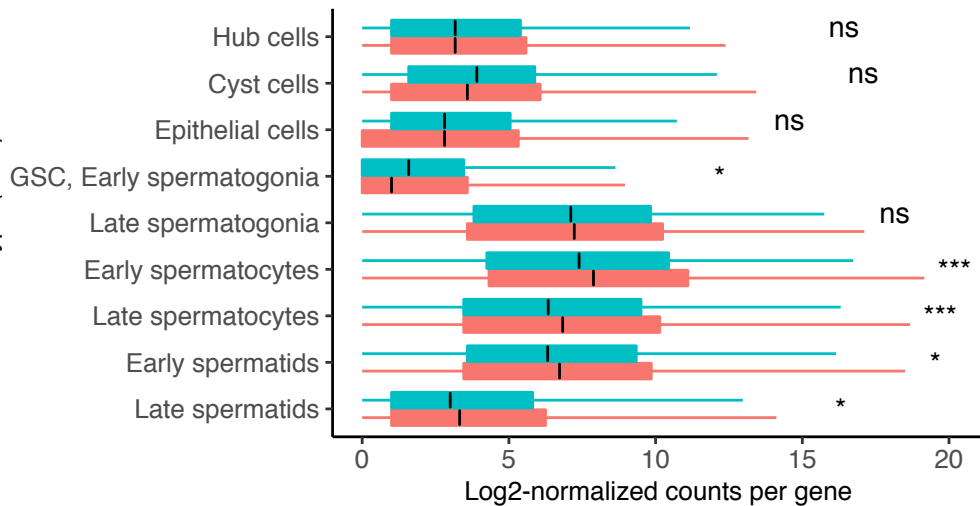

Chromosome

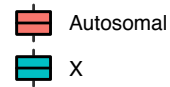

Supplement: S5 Fig — Corresponding to Fig 2, both strains support the appearance of pre-meiotic dosage compensation, somatic dosage compensation, and meiotic and post-meiotic X downregulation. In addition, in both strains, GSC and early spermatogonia appear to show X over-compensation. P values are from a two-tailed Wilcoxon test of the null hypothesis that X and autosomal counts are equal, adjusted with Holm’s correction. (PDF) [file pgen.1009728.s005.pdf]

**A**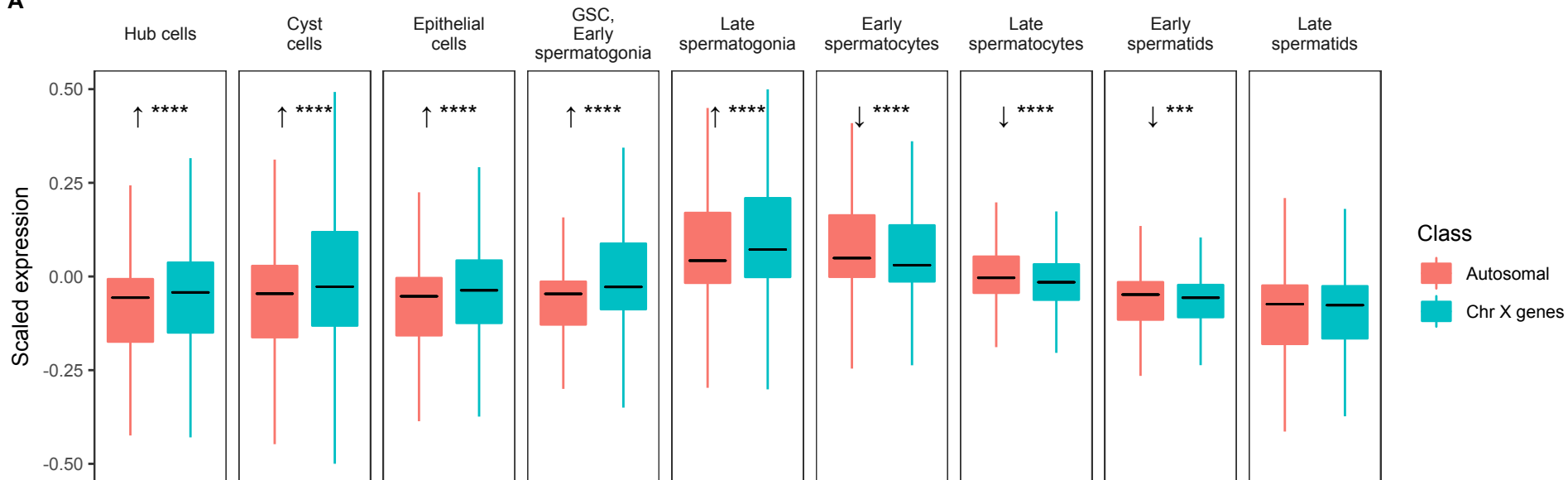**B**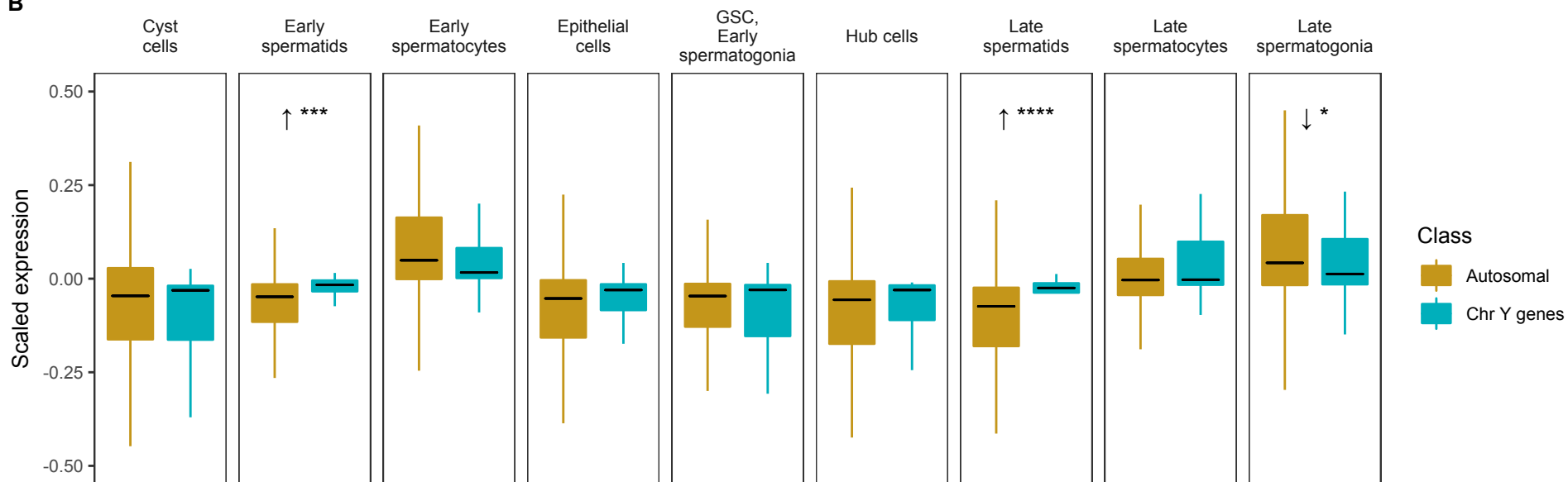

Supplement: S6 Fig — A) Boxplots indicate the distribution of scaled expression of autosomal and X chromosome genes within each cell type. 0 represents a gene’s mean expression across all cell types. In hub cells, epithelial cells, and premeiotic germ cells, scaled expression of X genes exceeds that of autosomal genes, suggesting that these cells experience X chromosome dosage compensation. Asterisks represent Holm-adjusted p values of directional Wilcoxon tests. B) Scaled expression of Y chromosome genes exceeds that of the autosomes in late spermatocytes, early spermatids, and late spermatids. This indicates that after meiosis, Y chromosome genes are not downregulated to the same extent as autosomal genes. Asterisks represent p values as follows: ns: >0.05, *<0.05, **<0.005, ***<0.0005, ****<0.00005. (PDF) [file pgen.1009728.s006.pdf]

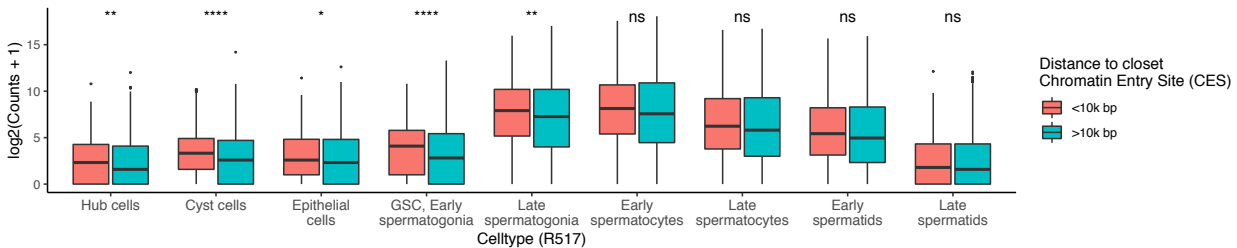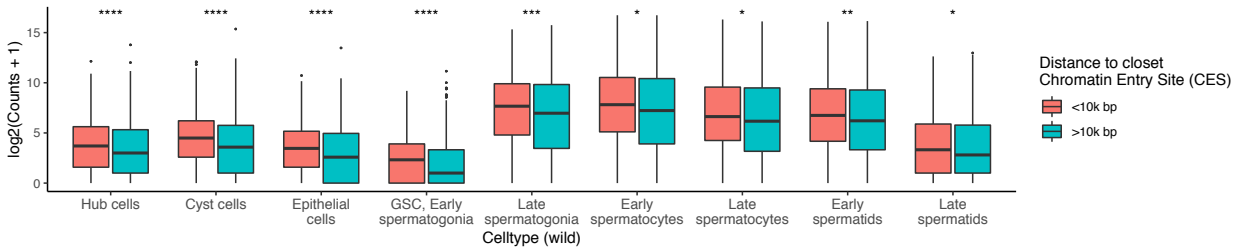

Supplement: S7 Fig — Both datasets agree that somatic and pre-meiotic cells have a statistical enrichment of counts detected from genes within 10000 bp from a CES. In the wild dataset, however, proximal genes are enriched in every cell type, although less so in meiotic and post-meiotic cells than in pre-meiotic and somatic cells. (PDF) [file pgen.1009728.s007.pdf]

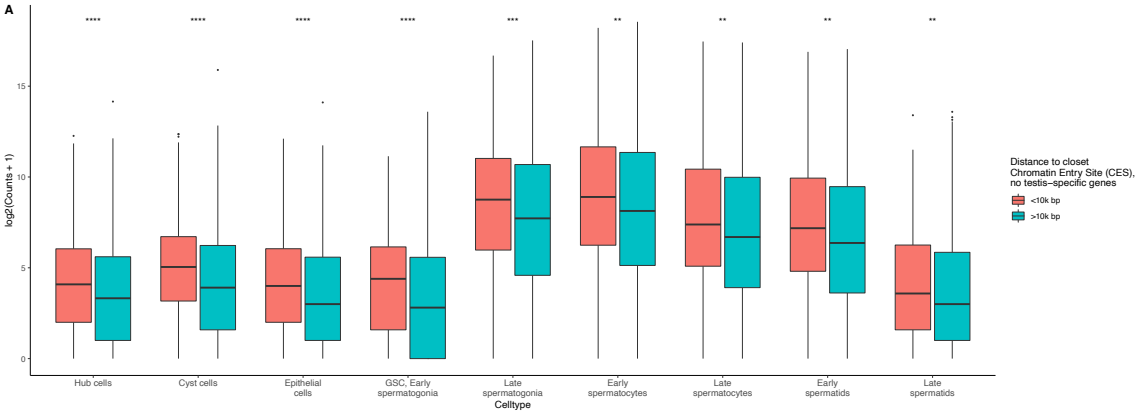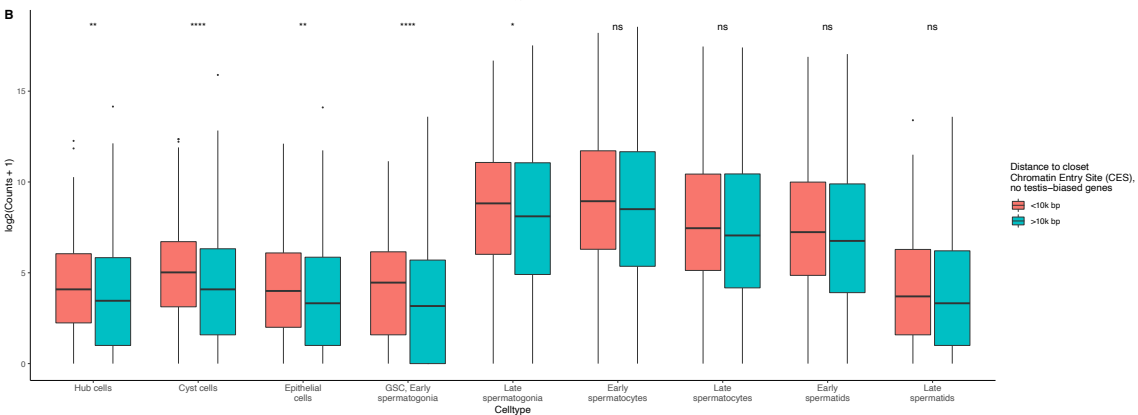

Supplement: S8 Fig — Shown is the analysis from Fig 3, repeated with testis-specific or testis-biased genes removed from the analysis. Neither result changes the conclusions in Fig 3. (PDF) [file pgen.1009728.s008.pdf]

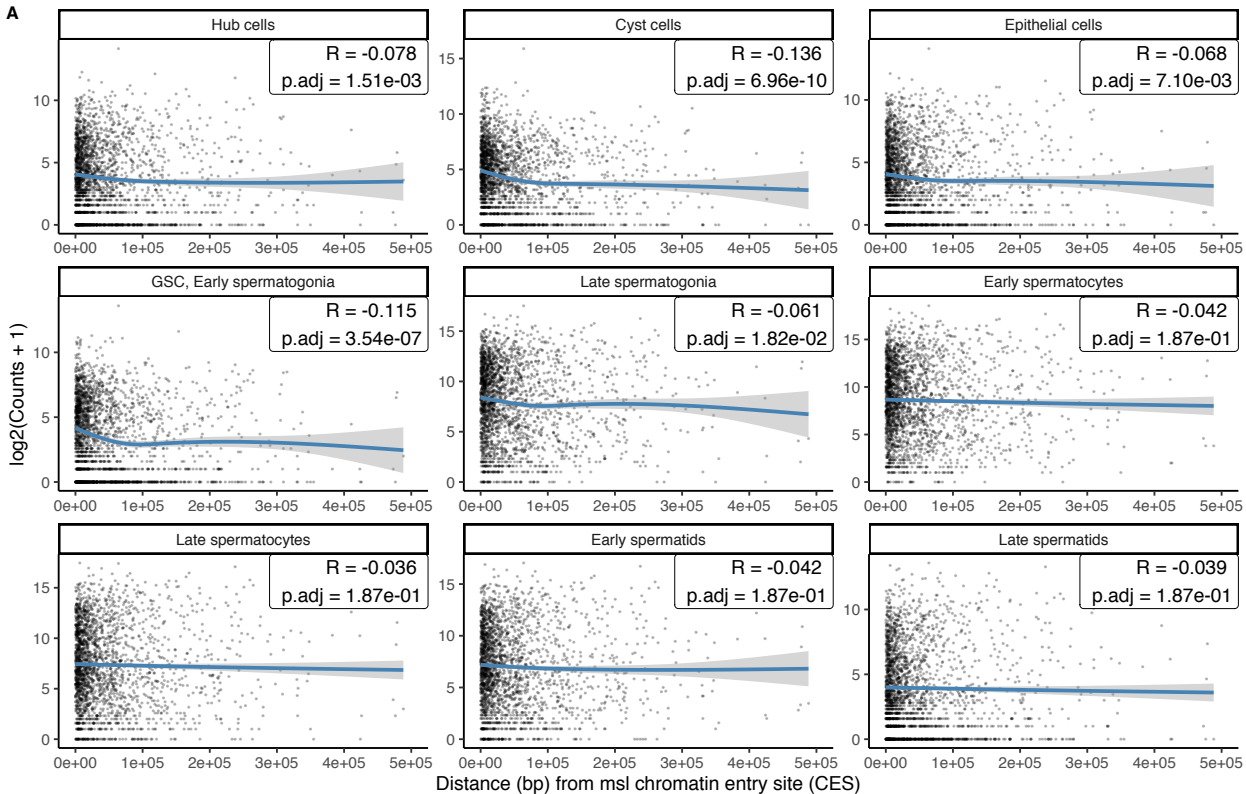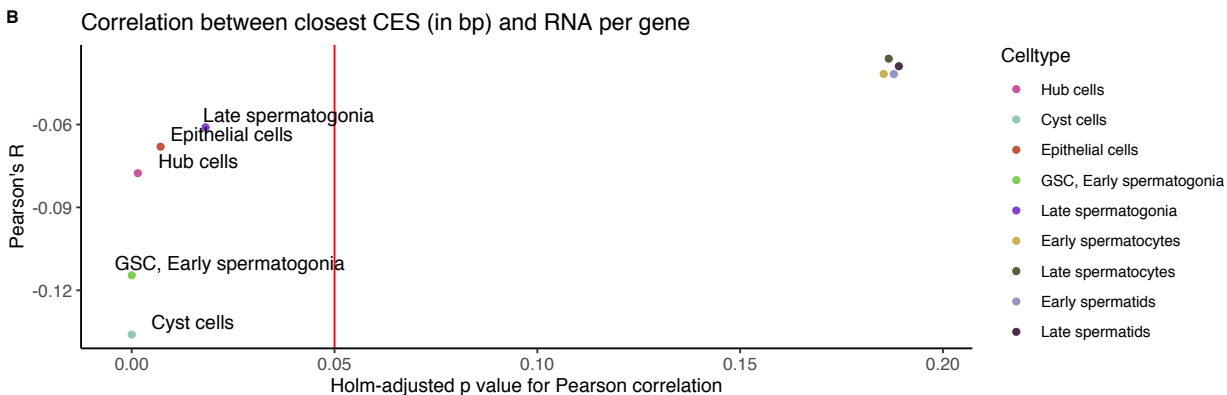

Supplement: S9 Fig — A) Each dot is an X chromosome gene; the X axis is the distance (in bp) between the gene start and the closest Chromatin Entry Site (CES) from Alekseyenko et al. 2008. The Y axis is the log-transformed sum of all counts of that gene in a cell type for every gene. The black line is a Loess regression showing an approximate trend between the two axes. B) Pearson’s R shows that CES distance loosely correlates with RNA counts in hub, cyst, epithelial, GSC, early spermatogonia, and late spermatogonia cells, all cell types where we found evidence of dosage compensation. Spermatocytes and spermatids have Pearson’s R closer to zero than DC-exhibiting cells, indicating less of a relationship between CES distance and transcription. In addition, these non-dosage-compensated cell types have a high Holm-adjusted p value, suggesting that distance and counts are not likely correlated in these cells. (PDF) [file pgen.1009728.s009.pdf]

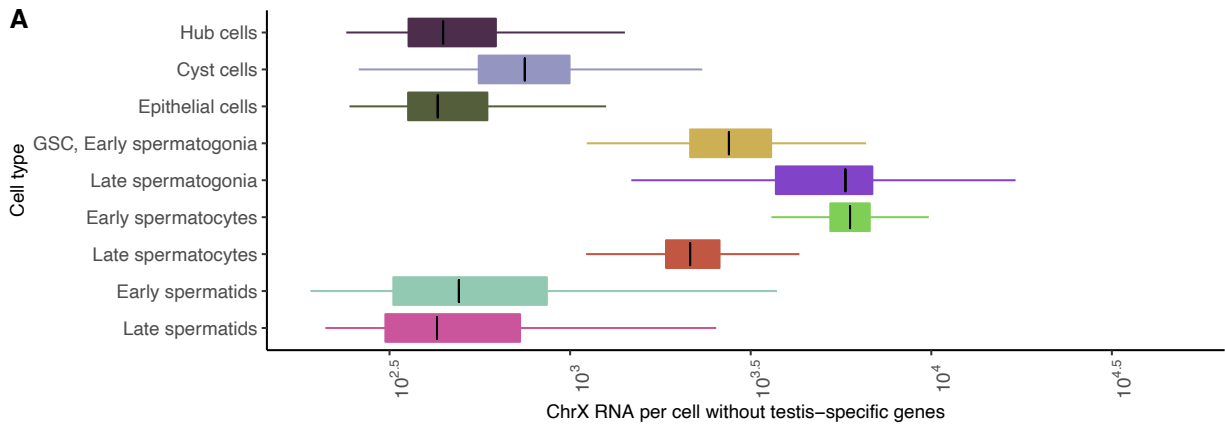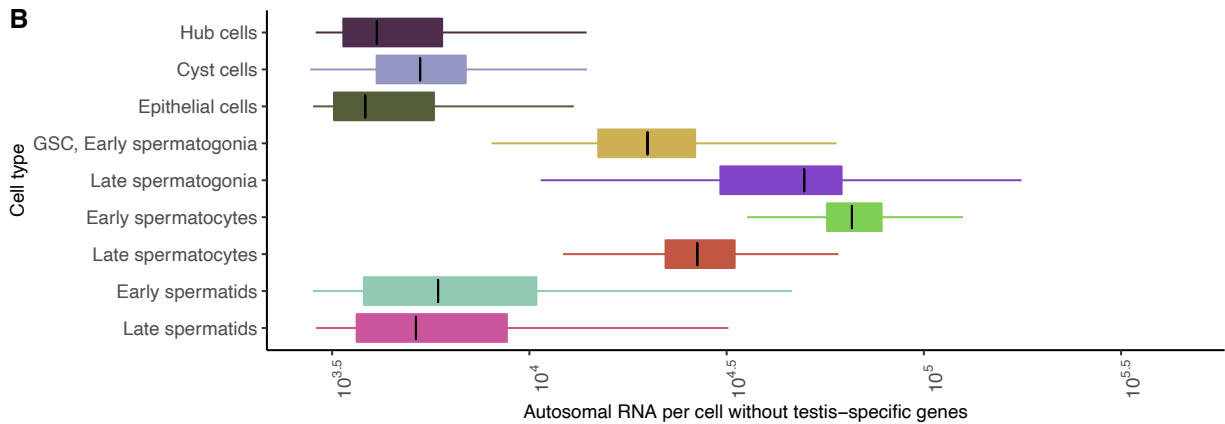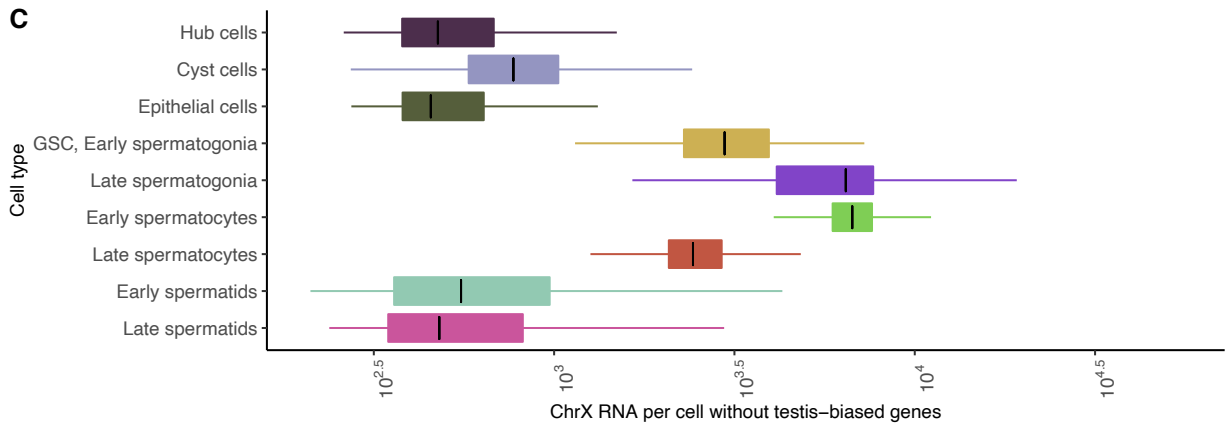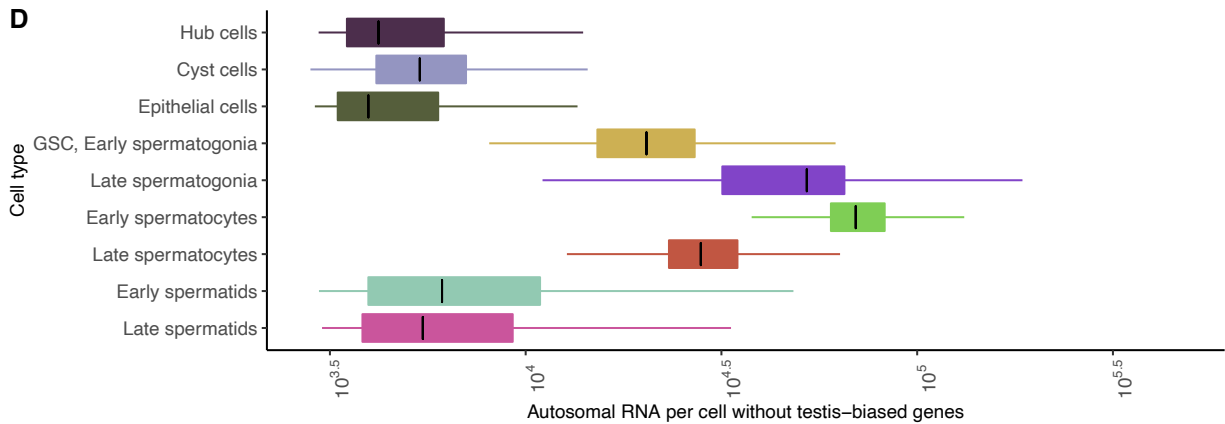

Supplement: S10 Fig — Each panel corresponds to Fig 1B, with testis-specific or testis-biased genes removed from the dataset. Overall patterns of RNA per cell do not significantly change. (PDF) [file pgen.1009728.s010.pdf]

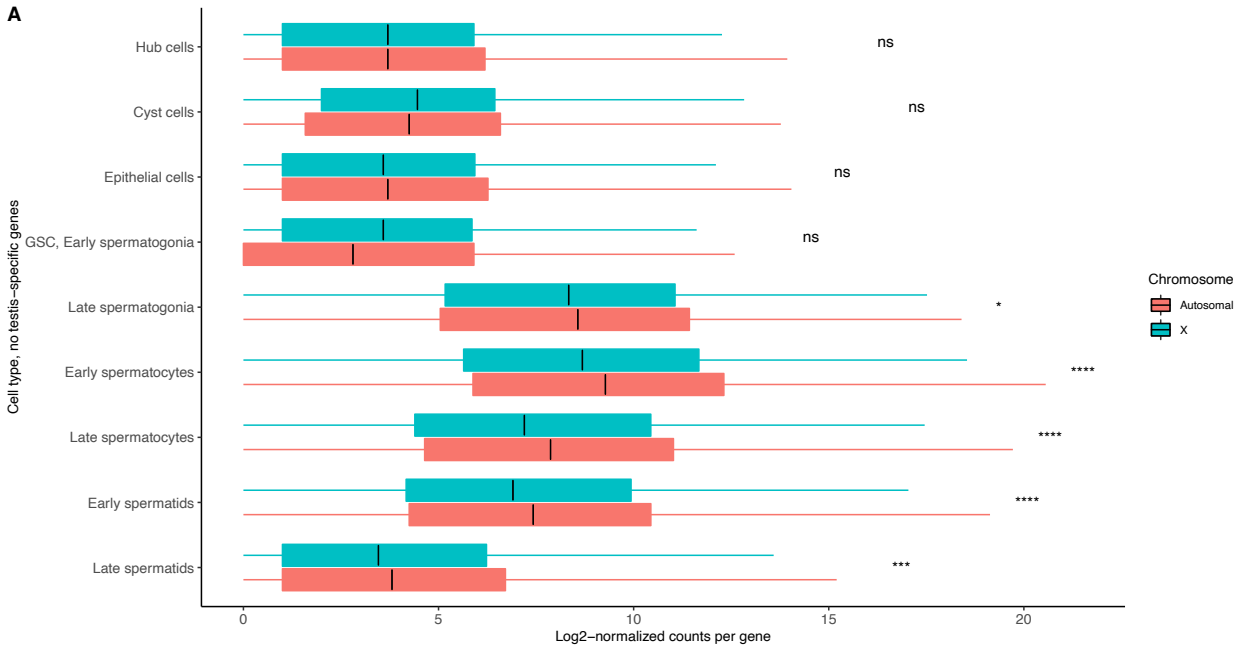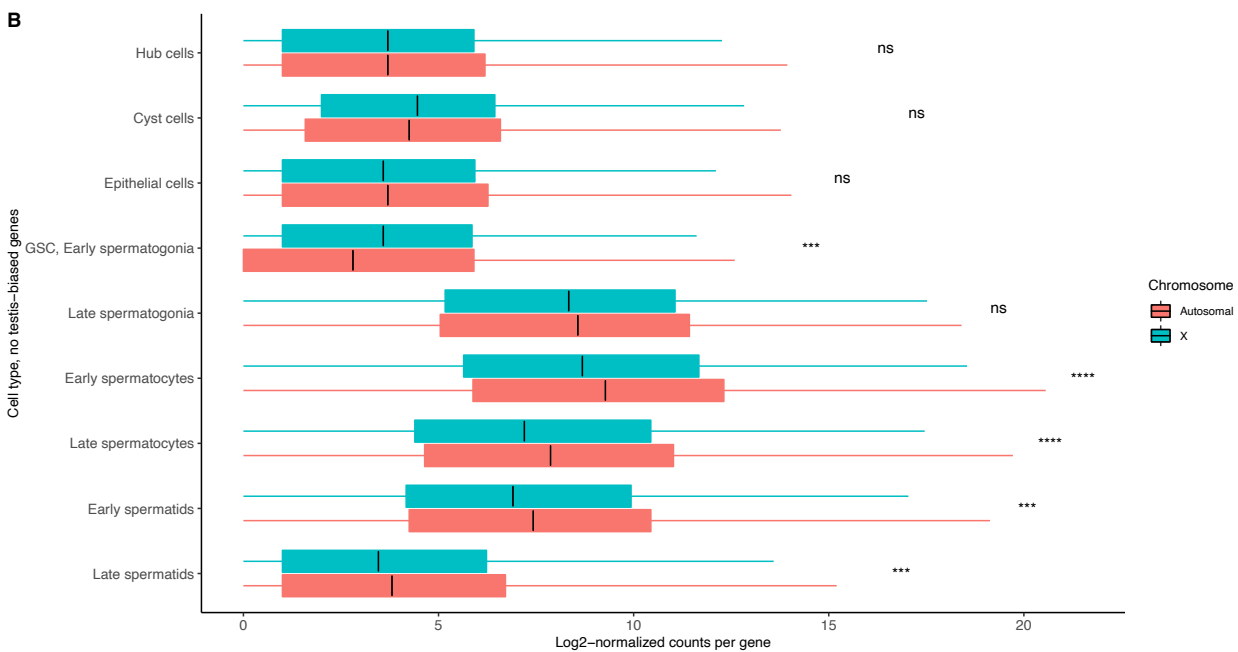

Supplement: S11 Fig — This is the same analysis as Fig 2, with testis-specific genes removed. The overall patterns of pre-meiotic dosage compensation and meiotic X downregulation are preserved, indicating that these genes did not influence our main findings. One difference from Fig 2 is that when testis-specific genes are removed, there is no longer statistical enrichment of X chromosome genes in GSC and early spermatogonia, indicating that testis-specific genes contribute to the appearance of X-chromosome overcompensation in these cells. Another difference is that without testis-biased genes, late spermatogonia no longer show statistical depletion of X chromosome counts, suggesting that these genes contribute to the earliest signs of reduced dosage compensation. (PDF) [file pgen.1009728.s011.pdf]

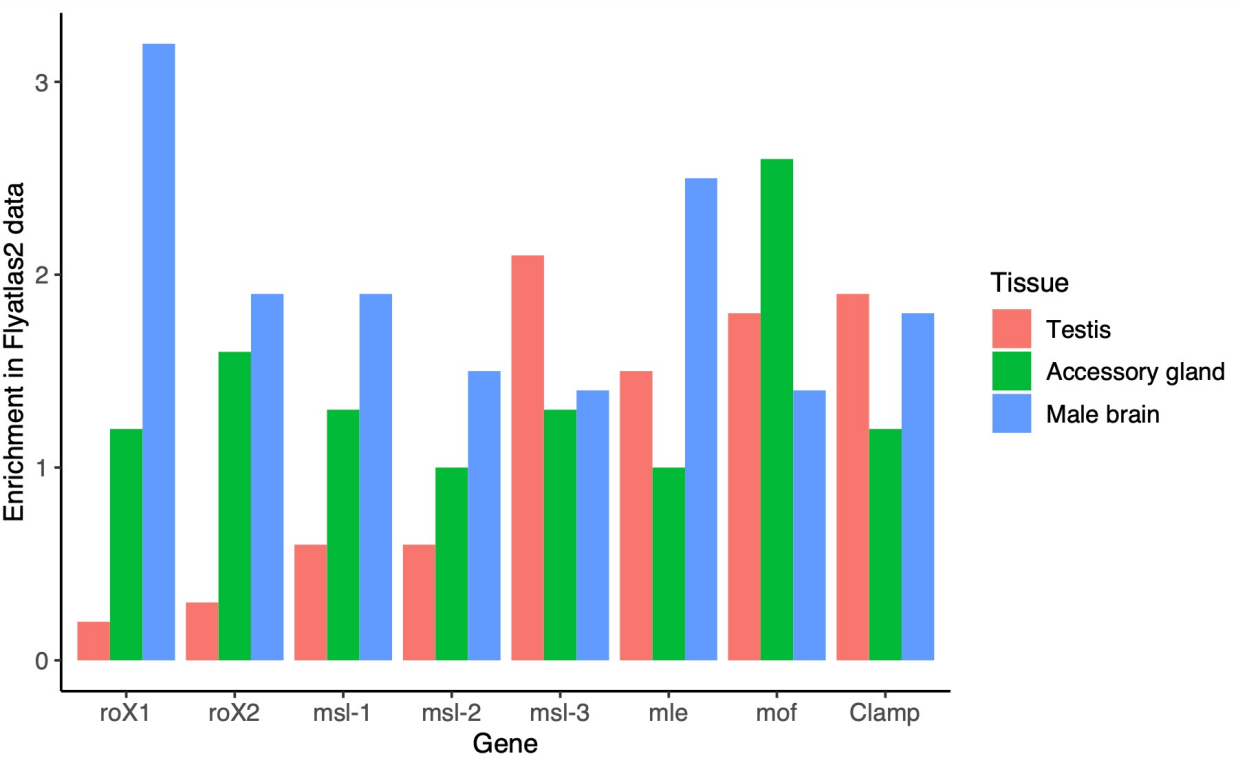

Supplement: S12 Fig — For each gene in the DCC, we queried the FlyAtlas2 website for their calculated enrichments in selected tissues compared to all tissues. RoX1, roX2, msl-1 and msl-2 are very depleted in testis compared to somatic tissues, corresponding to their relatively stochastic expression in our scRNA-seq and RNA-FISH data. (PDF) [file pgen.1009728.s012.pdf]

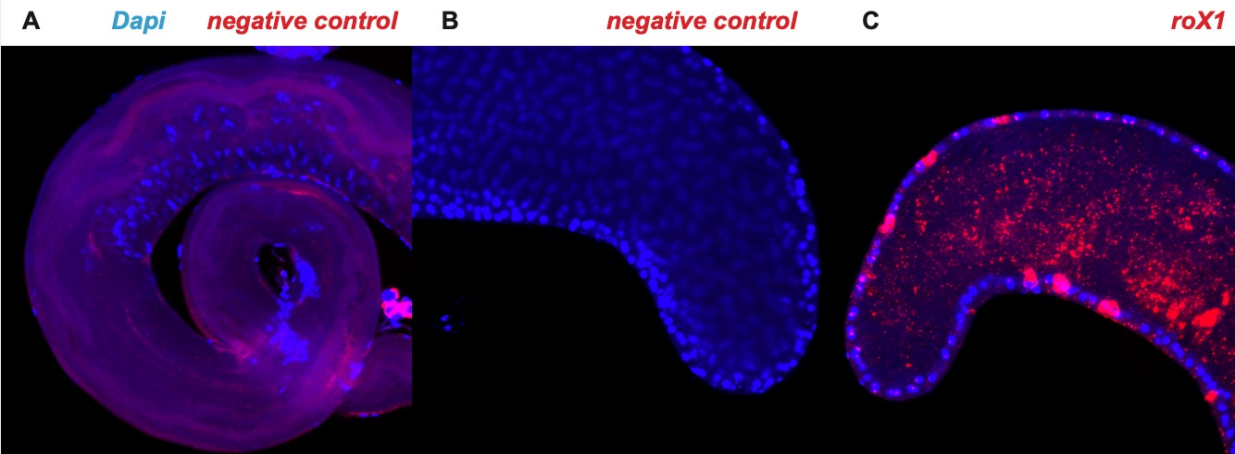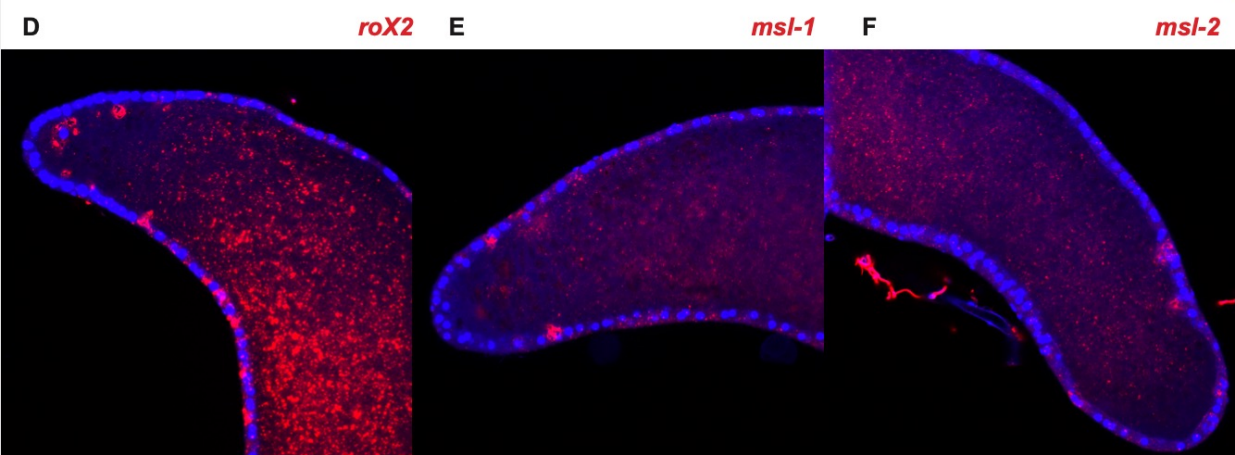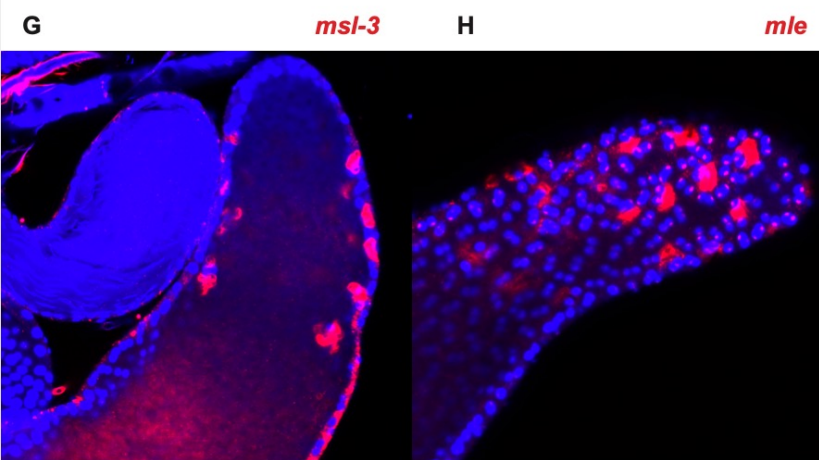

Supplement: S13 Fig — A) roX1 antisense negative control in testis. B) roX1 antisense negative control in the accessory gland. C) roX1 shows nuclear expression in accessory gland main cells and diffuse puncta in accessory gland lumen, as well as membrane expression in secondary cells. D) In the accessory gland, small patches of roX2 are expressed in the cytoplasm and nuclei of main cells and along membranes of secondary cells. roX2 is prevalent in accessory gland lumen as diffuse foci. E) mle is expressed as dense foci localized in nuclei of main cells, with smaller foci in the cytoplasm of main cells. In secondary cells, it is enriched near membranes and shows smaller and fewer dots in the accessory gland lumen. F) msl-2 shows small foci distributed at low levels in main cell cytoplasm. In secondary cells, it is enriched along membranes and in discrete foci in the accessory gland lumen. G) msl-3 shows strong expression patterns in the cytoplasm of main cells and the membranes of secondary cells, with smaller diffuse foci distributed in the accessory gland lumen. (PDF) [file pgen.1009728.s013.pdf]
