# Supplementary material for: Single-cell RNA-sequencing reveals pre-meiotic X-chromosome dosage compensation in Drosophila testis
Source: PLoS Genet. 2021 Aug 17;17(8):e1009728. doi: 10.1371/journal.pgen.1009728 (PMC8396764; doi:10.1371/journal.pgen.1009728)
Supplement: S1 Table — The X chromosome is most highly transcribed compared to autosomes in GSC and spermatogonia. (DOCX) [file pgen.1009728.s014.docx]

| Cell type | X counts | Autosome counts | X/autosome total count ratio |
| --- | --- | --- | --- |
| Hub cells | 662 | 6349 | 0.104 |
| Cyst cells | 895 | 6647 | 0.135 |
| Epithelial | 904 | 8669 | 0.104 |
| GSC, early spermatogonia | 3263 | 22448 | 0.145 |
| Late spermatogonia | 5979 | 49936 | 0.120 |
| Early spermatocytes | 6804 | 71689 | 0.095 |
| Late spermatocytes | 2749 | 31699 | 0.087 |
| Early spermatids | 785 | 8678 | 0.090 |
| Late spermatids | 851 | 9677 | 0.088 |

**S1 Table: Mean counts per cell from the X chromosome and autosomes in every cell type, corresponding to Fig 1B**. The X chromosome is most highly transcribed compared to autosomes in GSC and spermatogonia.
