# Supplementary material for: Single-cell RNA-sequencing reveals pre-meiotic X-chromosome dosage compensation in Drosophila testis
Source: PLoS Genet. 2021 Aug 17;17(8):e1009728. doi: 10.1371/journal.pgen.1009728 (PMC8396764; doi:10.1371/journal.pgen.1009728)
Supplement: S2 Table — Corresponding to S9 Fig, this is the Pearson’s R between distance and log2(counts+1) for every gene per cell type, with Holm-adjusted p values. Cell types with evidence of dosage compensation are shown with an asterisk. (DOCX) [file pgen.1009728.s015.docx]

| Cell type | P value | Pearson’s R | Adjusted p value |
| --- | --- | --- | --- |
| Hub cells* | 2.15e-04 | 1.51e-03 | 1.51e-03 |
| Cyst cells* | 7.73e-11 | 6.96e-10 | 6.96e-10 |
| Epithelial cells* | 1.18e-03 | 7.10e-03 | 7.10e-03 |
| GSC, early spermatogonia* | 4.43e-08 | 3.54e-07 | 3.54e-07 |
| Late spermatogonia* | 3.64e-03 | 1.82e-02 | 1.82e-02 |
| Early spermatocytes | 4.71e-02 | 1.87e-01 | 1.87e-01 |
| Late spermatocytes | 8.48e-02 | 1.87e-01 | 1.87e-01 |
| Early spermatids | 4.68e-02 | 1.87e-01 | 1.87e-01 |
| Late spermatids | 6.37e-02 | 1.87e-01 | 1.87e-01 |

**S2 Table: Correlation between distance and transcription for X chromosome genes by cell type.** Corresponding to Supplemental figure 9, this is the Pearson’s R between distance and log2(counts+1) for every gene per cell type, with Holm-adjusted p values. Cell types with evidence of dosage compensation are shown with an asterisk.
