# Supplementary material for: Single-cell RNA-sequencing reveals pre-meiotic X-chromosome dosage compensation in Drosophila testis
Source: PLoS Genet. 2021 Aug 17;17(8):e1009728. doi: 10.1371/journal.pgen.1009728 (PMC8396764; doi:10.1371/journal.pgen.1009728)
Supplement: S3 Table — We have converted the start and end coordinates to FlyBase R6 version using the FlyBase coordinates converter. (DOCX) [file pgen.1009728.s016.docx]

| Table | dm5 start | dm5 end | dm5 max | R5 coordinates | R6 coordinates |
| --- | --- | --- | --- | --- | --- |
| 1 | 366390 | 367190 | 366919 | X:366,390..367,190 | X:472,357..473,157 |
| 2 | 546603 | 549803 | 548347 | X:546,603..549,803 | X:652,570..655,770 |
| 3 | 655425 | 657601 | 656386 | X:655,425..657,601 | X:761,392..763,568 |
| 4 | 691829 | 692929 | 692277 | X:691,829..692,929 | X:797,796..798,896 |
| 5 | 936445 | 938345 | 937160 | X:936,445..938,345 | X:1,042,412..1,044,312 |
| 6 | 1267881 | 1269481 | 1268824 | X:1,267,881..1,269,481 | X:1,373,848..1,375,448 |
| 7 | 1356309 | 1357009 | 1356654 | X:1,356,309..1,357,009 | X:1,462,276..1,462,976 |
| 8 | 1374493 | 1375994 | 1375633 | X:1,374,493..1,375,994 | X:1,480,460..1,481,961 |
| 9 | 1587060 | 1588360 | 1588018 | X:1,587,060..1,588,360 | X:1,693,027..1,694,327 |
| 10 | 1774480 | 1775880 | 1775057 | X:1,774,480..1,775,880 | X:1,880,447..1,881,847 |
| 11 | 1786722 | 1788324 | 1787736 | X:1,786,722..1,788,324 | X:1,892,689..1,894,291 |
| 12 | 1918163 | 1919433 | 1918996 | X:1,918,163..1,919,433 | X:2,024,130..2,025,400 |
| 13 | 1955928 | 1957528 | 1956479 | X:1,955,928..1,957,528 | X:2,061,895..2,063,495 |
| 14 | 2072201 | 2074301 | 2072978 | X:2,072,201..2,074,301 | X:2,178,168..2,180,268 |
| 15 | 2125312 | 2126612 | 2125856 | X:2,125,312..2,126,612 | X:2,231,279..2,232,579 |
| 16 | 2236530 | 2238455 | 2237633 | X:2,236,530..2,238,455 | X:2,342,497..2,344,422 |
| 17 | 2339621 | 2341321 | 2340260 | X:2,339,621..2,341,321 | X:2,445,588..2,447,288 |
| 18 | 2492710 | 2493672 | 2493035 | X:2,492,710..2,493,672 | X:2,598,677..2,599,639 |
| 19 | 2520597 | 2521797 | 2521053 | X:2,520,597..2,521,797 | X:2,626,564..2,627,764 |
| 20 | 2628439 | 2629239 | 2628637 | X:2,628,439..2,629,239 | X:2,734,406..2,735,206 |
| 21 | 3375765 | 3377065 | NA | X:3,375,765..3,377,065 | X:3,481,732..3,483,032 |
| 22 | 3694068 | 3695885 | 3694893 | X:3,694,068..3,695,885 | X:3,800,035..3,801,852 |
| 23 | 3684985 | 3686792 | 3685816 | X:3,684,985..3,686,792 | X:3,790,952..3,792,759 |
| 24 | 3753268 | 3756637 | 3755452 | X:3,753,268..3,756,637 | X:3,859,235..3,862,604 |
| 25 | 3843793 | 3844593 | NA | X:3,843,793..3,844,593 | X:3,949,760..3,950,560 |
| 26 | 4020713 | 4022091 | 4021840 | X:4,020,713..4,022,091 | X:4,126,680..4,128,058 |
| 27 | 4438063 | 4439388 | 4439145 | X:4,438,063..4,439,388 | X:4,544,030..4,545,355 |
| 28 | 4578089 | 4578789 | 4578482 | X:4,578,089..4,578,789 | X:4,684,056..4,684,756 |
| 29 | 4598677 | 4600577 | 4599396 | X:4,598,677..4,600,577 | X:4,704,644..4,706,544 |
| 30 | 4969274 | 4970664 | 4970032 | X:4,969,274..4,970,664 | X:5,075,241..5,076,631 |
| 31 | 5308545 | 5310185 | 5309312 | X:5,308,545..5,310,185 | X:5,414,512..5,416,152 |
| 32 | 5563745 | 5565145 | 5564049 | X:5,563,745..5,565,145 | X:5,669,712..5,671,112 |
| 33 | 5653268 | 5654832 | 5653868 | X:5,653,268..5,654,832 | X:5,759,235..5,760,799 |
| 34 | 5681510 | 5684212 | 5682909 | X:5,681,510..5,684,212 | X:5,787,477..5,790,179 |
| 35 | 5804451 | 5806879 | 5806305 | X:5,804,451..5,806,879 | X:5,910,418..5,912,846 |
| 36 | 5976245 | 5977745 | 5977295 | X:5,976,245..5,977,745 | X:6,082,212..6,083,712 |
| 37 | 6122783 | 6123483 | NA | X:6,122,783..6,123,483 | X:6,228,750..6,229,450 |
| 38 | 6125383 | 6126429 | 6126019 | X:6,125,383..6,126,429 | X:6,231,350..6,232,396 |
| 39 | 6172809 | 6173997 | 6173582 | X:6,172,809..6,173,997 | X:6,278,776..6,279,964 |
| 40 | 6264168 | 6265668 | 6265067 | X:6,264,168..6,265,668 | X:6,370,135..6,371,635 |
| 41 | 6431763 | 6432463 | 6431910 | X:6,431,763..6,432,463 | X:6,537,730..6,538,430 |
| 42 | 6563384 | 6564901 | 6564053 | X:6,563,384..6,564,901 | X:6,669,351..6,670,868 |
| 43 | 6705400 | 6706845 | 6706224 | X:6,705,400..6,706,845 | X:6,811,367..6,812,812 |
| 44 | 7181094 | 7183678 | 7182556 | X:7,181,094..7,183,678 | X:7,287,061..7,289,645 |
| 45 | 7213876 | 7217359 | 7215106 | X:7,213,876..7,217,359 | X:7,319,843..7,323,326 |
| 46 | 7618472 | 7619472 | 7619172 | X:7,618,472..7,619,472 | X:7,724,439..7,725,439 |
| 47 | 7790451 | 7792008 | 7790905 | X:7,790,451..7,792,008 | X:7,896,418..7,897,975 |
| 48 | 7942825 | 7943937 | 7943529 | X:7,942,825..7,943,937 | X:8,048,792..8,049,904 |
| 49 | 7981841 | 7982605 | 7982237 | X:7,981,841..7,982,605 | X:8,087,808..8,088,572 |
| 50 | 8028174 | 8029974 | 8029113 | X:8,028,174..8,029,974 | X:8,134,141..8,135,941 |
| 51 | 8142104 | 8143004 | 8142580 | X:8,142,104..8,143,004 | X:8,248,071..8,248,971 |
| 52 | 8297145 | 8299963 | 8298407 | X:8,297,145..8,299,963 | X:8,403,112..8,405,930 |
| 53 | 8582587 | 8584087 | 8583332 | X:8,582,587..8,584,087 | X:8,688,554..8,690,054 |
| 54 | 8799008 | 8800137 | 8799773 | X:8,799,008..8,800,137 | X:8,904,975..8,906,104 |
| 55 | 9038744 | 9039544 | 9039023 | X:9,038,744..9,039,544 | X:9,144,711..9,145,511 |
| 56 | 9224784 | 9226114 | 9225778 | X:9,224,784..9,226,114 | X:9,330,751..9,332,081 |
| 57 | 9446480 | 9447780 | 9447253 | X:9,446,480..9,447,780 | X:9,552,447..9,553,747 |
| 58 | 9578393 | 9581795 | 9580787 | X:9,578,393..9,581,795 | X:9,684,360..9,687,762 |
| 59 | 9769835 | 9770835 | 9770616 | X:9,769,835..9,770,835 | X:9,875,802..9,876,802 |
| 60 | 9966323 | 9967023 | 9966601 | X:9,966,323..9,967,023 | X:10,072,290..10,072,990 |
| 61 | 10118520 | 10119764 | 10118946 | X:10,118,520..10,119,764 | X:10,224,487..10,225,731 |
| 62 | 10276207 | 10277160 | 10276600 | X:10,276,207..10,277,160 | X:10,382,174..10,383,127 |
| 63 | 10357313 | 10358390 | 10357608 | X:10,357,313..10,358,390 | X:10,463,280..10,464,357 |
| 64 | 10367323 | 10368423 | 10368421 | X:10,367,323..10,368,423 | X:10,473,290..10,474,390 |
| 65 | 10665610 | 10666474 | 10665977 | X:10,665,610..10,666,474 | X:10,771,577..10,772,441 |
| 66 | 10745605 | 10746405 | 10745748 | X:10,745,605..10,746,405 | X:10,851,572..10,852,372 |
| 67 | 10758754 | 10760630 | 10759843 | X:10,758,754..10,760,630 | X:10,864,721..10,866,597 |
| 68 | 10815961 | 10816662 | NA | X:10,815,961..10,816,662 | X:10,921,928..10,922,629 |
| 69 | 11038326 | 11039763 | 11039116 | X:11,038,326..11,039,763 | X:11,144,293..11,145,730 |
| 70 | 11057400 | 11058524 | 11057415 | X:11,057,400..11,058,524 | X:11,163,367..11,164,491 |
| 71 | 11290632 | 11293111 | 11291584 | X:11,290,632..11,293,111 | X:11,396,599..11,399,078 |
| 72 | 11293774 | 11294576 | NA | X:11,293,774..11,294,576 | X:11,399,741..11,400,543 |
| 73 | 11472953 | 11474927 | 11474270 | X:11,472,953..11,474,927 | X:11,578,920..11,580,894 |
| 74 | 11595401 | 11598135 | 11595759 | X:11,595,401..11,598,135 | X:11,701,368..11,704,102 |
| 75 | 11616668 | 11617791 | 11617352 | X:11,616,668..11,617,791 | X:11,722,635..11,723,758 |
| 76 | 11722160 | 11723229 | 11722824 | X:11,722,160..11,723,229 | X:11,828,127..11,829,196 |
| 77 | 11758511 | 11761239 | 11760360 | X:11,758,511..11,761,239 | X:11,864,478..11,867,206 |
| 78 | 11904033 | 11906110 | 11905008 | X:11,904,033..11,906,110 | X:12,010,000..12,012,077 |
| 79 | 12542747 | 12545401 | 12544973 | X:12,542,747..12,545,401 | X:12,648,714..12,651,368 |
| 80 | 12604385 | 12605085 | 12604739 | X:12,604,385..12,605,085 | X:12,710,352..12,711,052 |
| 81 | 12609483 | 12610183 | 12610103 | X:12,609,483..12,610,183 | X:12,715,450..12,716,150 |
| 82 | 12646261 | 12647087 | NA | X:12,646,261..12,647,087 | X:12,752,228..12,753,054 |
| 83 | 12653827 | 12656232 | 12654565 | X:12,653,827..12,656,232 | X:12,759,794..12,762,199 |
| 84 | 12804471 | 12806996 | 12805027 | X:12,804,471..12,806,996 | X:12,910,438..12,912,963 |
| 85 | 13093353 | 13096385 | 13095036 | X:13,093,353..13,096,385 | X:13,199,320..13,202,352 |
| 86 | 13157101 | 13157829 | 13157555 | X:13,157,101..13,157,829 | X:13,263,068..13,263,796 |
| 87 | 13234896 | 13237464 | 13235886 | X:13,234,896..13,237,464 | X:13,340,863..13,343,431 |
| 88 | 13283549 | 13284273 | NA | X:13,283,549..13,284,273 | X:13,389,516..13,390,240 |
| 89 | 13314325 | 13315325 | 13315226 | X:13,314,325..13,315,325 | X:13,420,292..13,421,292 |
| 90 | 13630255 | 13631714 | 13630460 | X:13,630,255..13,631,714 | X:13,736,222..13,737,681 |
| 91 | 13664247 | 13665281 | 13664853 | X:13,664,247..13,665,281 | X:13,770,214..13,771,248 |
| 92 | 13720782 | 13721482 | 13721027 | X:13,720,782..13,721,482 | X:13,826,749..13,827,449 |
| 93 | 13889981 | 13891745 | 13890982 | X:13,889,981..13,891,745 | X:13,995,948..13,997,712 |
| 94 | 13996764 | 14008829 | 13998406 | X:13,996,764..14,008,829 | X:14,102,731..14,114,796 |
| 95 | 14010329 | 14012229 | 14011574 | X:14,010,329..14,012,229 | X:14,116,296..14,118,196 |
| 96 | 14720985 | 14723179 | 14722568 | X:14,720,985..14,723,179 | X:14,826,952..14,829,146 |
| 97 | 14944690 | 14947962 | 14946204 | X:14,944,690..14,947,962 | X:15,050,657..15,053,929 |
| 98 | 14979863 | 14981953 | 14980466 | X:14,979,863..14,981,953 | X:15,085,830..15,087,920 |
| 99 | 15477142 | 15479342 | 15478359 | X:15,477,142..15,479,342 | X:15,583,109..15,585,309 |
| 100 | 15623738 | 15625770 | 15624768 | X:15,623,738..15,625,770 | X:15,729,705..15,731,737 |
| 101 | 15693722 | 15696722 | 15695055 | X:15,693,722..15,696,722 | X:15,799,689..15,802,689 |
| 102 | 15728080 | 15729132 | 15728442 | X:15,728,080..15,729,132 | X:15,834,047..15,835,099 |
| 103 | 15754121 | 15755823 | 15754707 | X:15,754,121..15,755,823 | X:15,860,088..15,861,790 |
| 104 | 15769177 | 15770977 | 15769834 | X:15,769,177..15,770,977 | X:15,875,144..15,876,944 |
| 105 | 15889446 | 15891074 | 15890650 | X:15,889,446..15,891,074 | X:15,995,413..15,997,041 |
| 106 | 16167399 | 16168907 | 16168205 | X:16,167,399..16,168,907 | X:16,273,366..16,274,874 |
| 107 | 16203823 | 16204705 | 16204038 | X:16,203,823..16,204,705 | X:16,309,790..16,310,672 |
| 108 | 16250181 | 16251481 | 16250882 | X:16,250,181..16,251,481 | X:16,356,148..16,357,448 |
| 109 | 16455838 | 16456864 | NA | X:16,455,838..16,456,864 | X:16,561,805..16,562,831 |
| 110 | 16501012 | 16502877 | 16502037 | X:16,501,012..16,502,877 | X:16,606,979..16,608,844 |
| 111 | 16621477 | 16623077 | NA | X:16,621,477..16,623,077 | X:16,727,444..16,729,044 |
| 112 | 16689604 | 16692767 | 16692394 | X:16,689,604..16,692,767 | X:16,795,571..16,798,734 |
| 113 | 16775983 | 16776983 | 16776671 | X:16,775,983..16,776,983 | X:16,881,950..16,882,950 |
| 114 | 16969751 | 16971404 | 16970798 | X:16,969,751..16,971,404 | X:17,075,718..17,077,371 |
| 115 | 17030560 | 17033492 | 17031424 | X:17,030,560..17,033,492 | X:17,136,527..17,139,459 |
| 116 | 17048415 | 17049615 | 17048811 | X:17,048,415..17,049,615 | X:17,154,382..17,155,582 |
| 117 | 17178939 | 17180238 | 17179865 | X:17,178,939..17,180,238 | X:17,284,906..17,286,205 |
| 118 | 17538938 | 17540038 | NA | X:17,538,938..17,540,038 | X:17,644,905..17,646,005 |
| 119 | 17547893 | 17550393 | 17549607 | X:17,547,893..17,550,393 | X:17,653,860..17,656,360 |
| 120 | 17598986 | 17601152 | 17600087 | X:17,598,986..17,601,152 | X:17,704,953..17,707,119 |
| 121 | 17714956 | 17717075 | 17715734 | X:17,714,956..17,717,075 | X:17,820,923..17,823,042 |
| 122 | 17819963 | 17821239 | NA | X:17,819,963..17,821,239 | X:17,925,930..17,927,206 |
| 123 | 17987153 | 17988753 | 17987981 | X:17,987,153..17,988,753 | X:18,093,120..18,094,720 |
| 124 | 17991993 | 17992693 | 17992605 | X:17,991,993..17,992,693 | X:18,097,960..18,098,660 |
| 125 | 18268228 | 18269396 | 18268774 | X:18,268,228..18,269,396 | X:18,374,195..18,375,363 |
| 126 | 18388748 | 18390439 | 18389692 | X:18,388,748..18,390,439 | X:18,494,715..18,496,406 |
| 127 | 18547720 | 18549220 | 18548520 | X:18,547,720..18,549,220 | X:18,653,687..18,655,187 |
| 128 | 18683463 | 18685163 | 18684116 | X:18,683,463..18,685,163 | X:18,789,430..18,791,130 |
| 129 | 18742623 | 18744205 | 18743223 | X:18,742,623..18,744,205 | X:18,848,590..18,850,172 |
| 130 | 18781253 | 18782810 | 18782227 | X:18,781,253..18,782,810 | X:18,887,220..18,888,777 |
| 131 | 19089662 | 19091279 | 19090428 | X:19,089,662..19,091,279 | X:19,195,629..19,197,246 |
| 132 | 19166379 | 19167579 | 19167145 | X:19,166,379..19,167,579 | X:19,272,346..19,273,546 |
| 133 | 19383500 | 19384800 | 19384312 | X:19,383,500..19,384,800 | X:19,489,467..19,490,767 |
| 134 | 19471825 | 19473867 | 19473298 | X:19,471,825..19,473,867 | X:19,577,792..19,579,834 |
| 135 | 19518350 | 19519250 | 19518692 | X:19,518,350..19,519,250 | X:19,624,317..19,625,217 |
| 136 | 19534337 | 19535866 | 19535151 | X:19,534,337..19,535,866 | X:19,640,304..19,641,833 |
| 137 | 19582304 | 19584399 | 19583395 | X:19,582,304..19,584,399 | X:19,688,271..19,690,366 |
| 138 | 19624437 | 19625391 | NA | X:19,624,437..19,625,391 | X:19,730,404..19,731,358 |
| 139 | 19635080 | 19635856 | 19635374 | X:19,635,080..19,635,856 | X:19,741,047..19,741,823 |
| 140 | 19752914 | 19753814 | 19753490 | X:19,752,914..19,753,814 | X:19,858,881..19,859,781 |
| 141 | 19917716 | 19919805 | 19918722 | X:19,917,716..19,919,805 | X:20,023,683..20,025,772 |
| 142 | 20052603 | 20053719 | 20053190 | X:20,052,603..20,053,719 | X:20,158,570..20,159,686 |
| 143 | 20102872 | 20103676 | NA | X:20,102,872..20,103,676 | X:20,231,798..20,232,602 |
| 144 | 20282194 | 20283326 | 20282867 | X:20,282,194..20,283,326 | X:20,411,167..20,412,299 |
| 145 | 20920307 | 20923420 | 20921199 | X:20,920,307..20,923,420 | X:21,049,280..21,052,393 |
| 146 | 21102348 | 21103548 | 21103032 | X:21,102,348..21,103,548 | X:21,231,321..21,232,521 |
| 147 | 21187950 | 21189650 | 21188853 | X:21,187,950..21,189,650 | X:21,316,923..21,318,623 |
| 148 | 21239454 | 21241240 | 21240544 | X:21,239,454..21,241,240 | X:21,368,427..21,370,213 |
| 149 | 21869967 | 21871175 | 21870626 | X:21,869,967..21,871,175 | X:22,468,131..22,469,339 |
| 150 | 21936497 | 21938643 | 21937487 | X:21,936,497..21,938,643 | X:22,534,661..22,536,807 |

**S3 Table: Chromatin entry sites from Alekseyenko et. al 2008 (DOI: 10.1016/j.cell.2008.06.033)**. We have converted the start and end coordinates to FlyBase R6 version using the FlyBase coordinates converter.
