# Supplementary material for: Single-cell RNA-sequencing reveals pre-meiotic X-chromosome dosage compensation in Drosophila testis
Source: PLoS Genet. 2021 Aug 17;17(8):e1009728. doi: 10.1371/journal.pgen.1009728 (PMC8396764; doi:10.1371/journal.pgen.1009728)
Supplement: S3 Fig — This corresponds to S2 Fig. Marker enrichment in both strains corroborates cell type assignments. (PDF) [file pgen.1009728.s003.pdf]

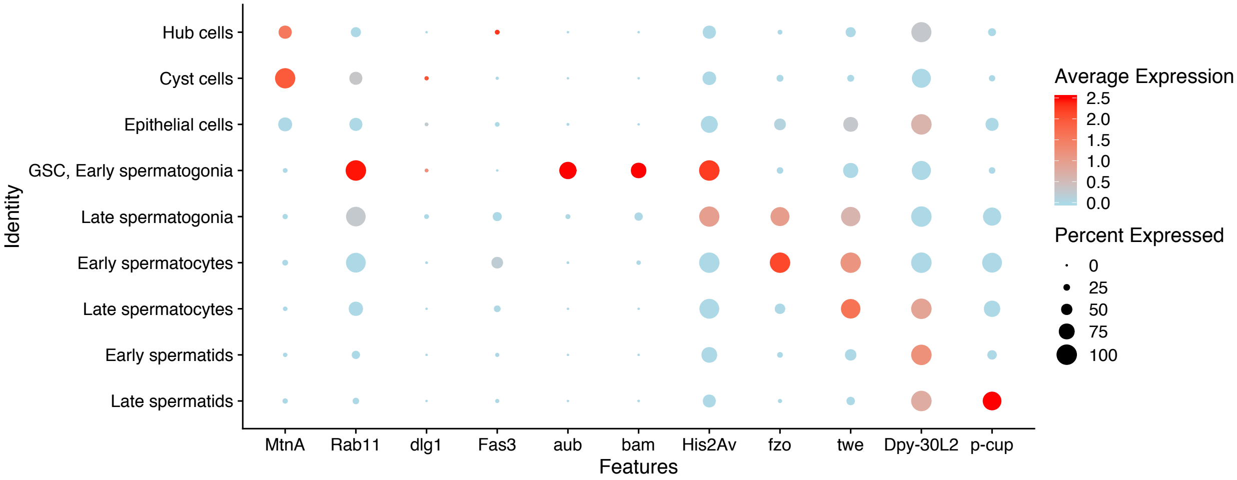

Hub cells
Cyst cells
Epithelial cells
GSC, Early spermatogonia
Late spermatogonia
Early spermatocytes
Late spermatocytes
Early spermatids
Late spermatids
Identity
MtnA
Rab11
dlg1
Fas3
aub
bam
His2Av
fzo
twe
Dpy-30L2
p-cup
Features
Average Expression
2.5
2.0
1.5
1.0
0.5
0.0
Percent Expressed
0
25
50
75
100

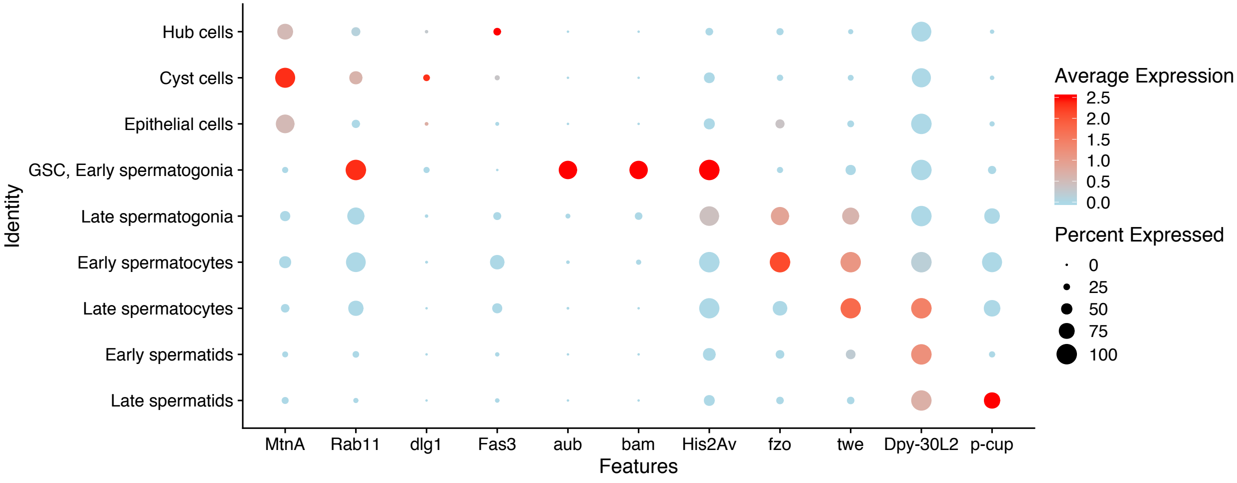

Hub cells
Cyst cells
Epithelial cells
GSC, Early spermatogonia
Late spermatogonia
Early spermatocytes
Late spermatocytes
Early spermatids
Late spermatids
Identity
MtnA
Rab11
dlg1
Fas3
aub
bam
His2Av
fzo
twe
Dpy-30L2
p-cup
Features
Average Expression
2.5
2.0
1.5
1.0
0.5
0.0
Percent Expressed
0
25
50
75
100
