# Supplementary material for: Single-cell RNA-sequencing reveals pre-meiotic X-chromosome dosage compensation in Drosophila testis
Source: PLoS Genet. 2021 Aug 17;17(8):e1009728. doi: 10.1371/journal.pgen.1009728 (PMC8396764; doi:10.1371/journal.pgen.1009728)
Supplement: Attachment — Submitted filename: Review.pdf [file pgen.1009728.s017.pdf]

Review of "Single-cell RNA-sequencing reveals pre-meiotic X-chromosome dosage compensation in Drosophila testes"

In this manuscript, the authors seek evidence for/against active dosage compensation in adult drosophila testes. They analyze an existing scRNA-seq dataset from the same group (Witt, et al. 2019). Although this manuscript presents: 1) a reanalysis of an existing dataset from the same authors, 2) confirmation in adult testes of a previously published observation in larval testes by Mahadevaraju et al. (2020), 3) confirmation of lack of MSL machinery in testes (Rastelli and Kuroda, 1998), <u>this is a creative use of an existing dataset during the covid pandemic that shut down many wet labs and I appreciate the novel perspective that is given by analysis of scRNA-seq data.</u>

The authors reanalyze the dataset from Witt et al., focusing specifically on X-linked genes in subpopulations of testes cells, finding evidence of dosage compensation in pre-meiotic and somatic cells, and evidence of excess "dosage compensation" in spermatogonia. They then break down the analysis into genes "close to" and "distant" from CE sites and find supportive evidence that confirms active dosage compensation in certain cell populations. Finally, they examine the expression of the dosage compensation machinery in testes by scRNA-seq and RNA FISH, finding little evidence of active canonical dosage compensation and instead suggesting that there exist alternative DC mechanisms. <u>Overall, this work is well written, the figures are nicely presented, and the authors make claims rooted in observation without drawing unnecessary extraordinary conclusions.</u>

I have only minor suggestions:

The authors have left CLAMP (which is an acronym and is therefore not "Clamp") out of Figure 4A. I believe this is a mistake, as it is referenced in the text. In the text, the authors should also justify why *clamp* RNA FISH is not included in their analysis. Note that I do not believe that *clamp* RNA FISH is a necessary addition to this manuscript, as evidence of *clamp* without the MSL machinery does not alter the conclusions, simply that the authors should justify this decision explicitly.

While RNA-FISH is appropriate for *roX1* and *roX2* lncRNAs, it is less conclusive for the DCC protein machinery, for which antibodies and tagged lines exist. However, these experiments have already been done, first by Rastelli and Kuroda (1998). The authors cite Rastelli, but I suggest that they add language directly comparing their RNA seq/FISH observations in Fig 4 to the Rastelli observations.

Please be explicit about what "negative control" means in figure legends (e.g. Sup Fig 13). In this figure legend there are also many acronyms (SC, MC, AG), which make it difficult to follow for those outside the immediate field.
