# Supplementary material for: Single-cell RNA-sequencing reveals pre-meiotic X-chromosome dosage compensation in Drosophila testis
Source: PLoS Genet. 2021 Aug 17;17(8):e1009728. doi: 10.1371/journal.pgen.1009728 (PMC8396764; doi:10.1371/journal.pgen.1009728)
Supplement: Attachment — Submitted filename: Response__Witt_etal_20210701.pdf [file pgen.1009728.s018.pdf]

**Response to comments**

**Response to editor's comments:**

The manuscript was fully evaluated at the editorial level and by independent peer reviewers. The reviewers appreciated the attention to an important problem, but raised some substantial concerns about the current manuscript.

**Response: We would like to thank the editor and reviewers for the extremely prompt, thoughtful, and fair feedback, and we have done our best to address the issues raised in this revised version.**

1. The current manuscript's use of "excess dosage compensation" should be clarified. Reviewer 3 states that the conclusion of overcompensation is likely due to the inclusion of X-linked testes-specific genes. Reviewer 2 also points out that, if the appearance of excess dosage compensation is due to gene-specific regulation, then it is not actually a form of dosage compensation at all. The claim of "excess dosage compensation" should be re-evaluated in light of the reviewer's comments. If the excess transcription on the X chromosome relative to autosomes in GSC/early spermatogonia is due to high expression of testes-specific genes, it is not accurate to refer to this phenomenon as "excess dosage compensation" and the manuscript should be revised accordingly. On the other hand, if gene-specific regulation does not explain the excess of transcription from the X chromosome, then the excess dosage phenomenon should be discussed further, as suggested by Reviewer 2 (points 9 & 12).

**Response: In the previous version, we have a section discussing the effect of X-linked testis-specific genes and dosage compensation. In this section, we found that after removing testis-specific genes, the excess dosage compensation is marginally insignificant (S11A Figure $p = 0.015$, $p.adj = 0.059$), which is in line with the notion from reviewer 2 and 3. However, if we remove testis-biased genes, excess dosage compensation pattern still exists. We think these results indicate that excess dosage compensation may exist to some extent. In the section, we reflect this thought as "Without these testis-specific genes, the distributions of X and autosome counts are similar, consistent with the degree of dosage compensation observed in somatic cells. This could indicate an uneven degree of dosage compensation for different gene classes in early germ cells. Whether the excessive up-regulation of testis-biased genes is through dosage compensation or other mechanism is unclear, however, it will be important to study the mechanism and the impact of this pattern in the future." However, we agree with the editor and the reviewers that we should tone down this result. We did the following work:**

**1) We deleted the sentence "GSC/early spermatogonia show evidence of excess dosage compensation (X:A = 1.83)" in the introduction, to make sure that the audiences do not treat this as a main message of the paper.**

**2) In the results section, when report these results, we immediately added a possible correlation with X-linked testis-specific genes: "In GSC and early spermatogonia, the X:autosome ratio increases to 1.83, suggestive of a possibility of excess dosage compensation (adjusted p value: 3.86e-04), although the pattern of excess dosage compensation might partly be caused by up-regulation of X-linked testis-specific genes (see below).".**

**3) We explained the results from S8, S10, and S11 figures (analysis after removing male-specific/biased genes) more clearly.**

**4) In the section "Testis-biased and testis-specific genes do not bias apparent dosage compensation" we discussed all possibilities.**

**5) We added a paragraph in the discussion (line 270-286) to reflect the above thoughts.**

2. clamp should be added to Figure 4A and a justification of why clamp was not used for RNA FISH should be included in the manuscript text. The RNA FISH results should also be explicitly compared to the immunostaining results from Rastelli & Kuroda (1998), as suggested by Reviewer 1.

**Response: We have added CLAMP to Figure 4A, and have clarified in the text that we focused our RNA-FISH experiments on *msl-1,2,3, mle,* and *mof,* the core components of the MSL complex. We also directly compared our results with the results from Rastelli & Kuroda (1998).**

**Response to reviewer 1:**

**"**The authors have left CLAMP (which is an acronym and is therefore not "Clamp") out of Figure 4A. I believe this is a mistake, as it is referenced in the text. In the text, the authors should also justify why clamp RNA FISH is not included in their analysis. Note that I do not believe that clamp RNA FISH is a necessary addition to this manuscript, as evidence of clamp without the MSL machinery does not alter the conclusions, simply that the authors should justify this decision explicitly."

**Response: Thank you for pointing out this omission. We have added CLAMP to Figure 4A, and have clarified in the text that we chose to focus our RNA-FISH experiments on *msl-1,2,3, mle,* and *mof,* the core components of the MSL complex.**

**As for the nomenclature of Clamp/CLAMP, we agree that the original authors who discovered CLAMP genes use capitalized word. FlyBase has recorded the gene symbol as "Clamp" even**

**though it is an abbreviation. To ensure consistency, when referencing the gene or RNA we chose to abide by FlyBase's standard, and when referencing the protein, we call it CLAMP.**

**"**The authors cite Rastelli, but I suggest that they add language directly comparing their RNA seq/FISH observations in Fig 4 to the Rastelli observations."

**Response: We have added more discussion of Rastelli and Kuroda (1998) in the Figure 4 legend, and to lines 218-220. We note that, of all the MSL proteins, they only found evidence of translation of MLE, and found that MLE does not localize to the X chromosome. Our results reported here is consistent with those of Rastelli and Kuroda (1998).**

"Please be explicit about what "negative control" means in figure legends (e.g. Sup Fig 13)."

**Response: For S13 figure, we have clarified that panels A and B are antisense RNA negative controls for *roX1* in testis and accessory gland. We have also removed all the abbreviations from this figure legend.**

**Response to reviewer 2:**

Reviewer #2: "…Some of the results presented in this manuscript have been reported previously (complete DC in soma, lack of DC in testis), but have been somewhat controversial. By using scRNA-seq, the authors are able to address these issues at a finer scale than previous studies that focused on whole tissues or dissected regions of tissues. Thus, I think they have generated valuable data that extend our knowledge of this topic. The data are available in a format convenient for researchers and should be an important resource. There is, however, a need for the authors to improve their presentation and expand upon some of their analyses."

**Response: We thank the positive comments for the manuscript and appreciate the comments and suggestions from the reviewer. We have addressed the reviewer's comments below and in the text.**

1. line 21: "X chromosome transcription is equalized in the somatic cells of both males and females" - not phrased clearly. Does DC equalise expression of X between sexes? Or between X and autosomes within males? The latter is what the authors investigate in the manuscript.

**Response: We revised the sentence. This line now reads: "Dosage compensation is a mechanism by which X chromosome transcription is equalized to that of autosomes in the somatic cells of both males and females."**

2. line 40: should be "needs"

**Response: This now reads: "Somatic expression of X-linked genes needs to be adjusted".**

3. line 51: authors don't define some abbreviations (DCC, MSL, MLE, MOF). Readers will probably be confused.

**Response: We have included the full names for these genes from lines 65-74.**

4. line 68: "Other work suggests that demasculinization of the X chromosome might be partly due to dosage compensation in Drosophila (Bachtrog et al., 2010)." - I think more explanation is needed here. Why would DC lead to demasculinization? One might expect the *absence* of DC to lead to demasculinisation.

**Response: The reviewer is right. Bachtrog et al., 2010 argues that male-biased X chromosome genes are away from dosage compensated regions. They argue that their results "are consistent with dosage compensation actively limiting or interfering with the evolution of male-biased gene expression at the X chromosome of Drosophila". We revised this sentence, and it now reads: "Other work found that male-biased X chromosome genes are usually found outside of dosage compensated regions, suggesting that dosage compensation influences patterns of sex-biased expression on X chromosome".**

5. lines 70-83: there are some awkward references to previous literature in which the researchers are mentioned by name apart from the citations, such as "Meiklejohn and Presgraves", "Rastelli & Kuroda", "Parsch group". It would be better to only cite the papers.

**Response: We originally wanted to highlight their contribution to the question. This has been changed to a neutral tone.**

6. line 75: "the mechanism of hypothetical germline DC is an additional mystery." How can there be a mechanism of something that is hypothetical?

**Response: This now reads: "Given that the MSL complex is thought not to localize to male germline X chromosomes [20], the mechanism of germline dosage compensation in *Drosophila* male germ cells remains a mystery [9]".**

7. line 82: "multiple transgenic insertions in X and autosomes made by the Parsch group (Hense et al., 2007; Kemkemer et al., 2011) show that X inactivation exceeds that expected for loss of dosage compensation" - this is true, but these transgene experiments also controlled reporter gene dose to always be one (whether on X or autosome), so gene dose or loss of DC can be ruled out as a cause for an expression difference. The same approach was used by Landeen et al. (PLoS Biol. 2016 Jul 12;14(7):e1002499).

**Response: Thank you for this insight. We have added new discussion of this point to lines 90-95, and have added this citation.**

8. Fig 1B: it might help to indicate which gene you used as a marker for each cell type in parentheses next to the names of the cell types on the figure.

**Response: We agree with this suggestion, and decided it worked better to add these gene symbols to figure 1A, since the marker gene figure was reported in Witt et al., 2019, although the datasets were different. The marker gene figure can be found in S2 and S3 supplemental Figure.**

9. Table 1: there seems to be a transition from "excess DC" in early spermatogonia to "DC" in late spermatogonia to "no DC" in early spermatocytes. Since this is a developmental progression, I am not sure about the conclusion in early spermatogonia. This is because the authors measure RNA content, but do not measure transcriptional activity directly. If there is an excess of X RNA in an early stage, then even if there is no DC in the next stage one might expect to see more X RNA simply because it remains from the previous stage and has not yet degraded. To me, the data seem to be in agreement with this interpretation. The unusual observation is that there is excess DC in GSC and early spermatogonia. After these stages, the results could be consistent with "no DC".

**Response: Thank you for this comment. We agree with the reviewer. For the excess dosage compensation results, please see our response above. For late spermatogonia we added a note on Table 1 to reflect your comments ". Late spermatogonia may also be interpreted as no DC if they contain most transcripts generated from early spermatogonia.". We added this sentence to lines 180-183: "The apparent dosage compensation in late spermatogonia could be caused by leftover transcripts from the surge of X chromosome RNA found in GSC/early spermatogonia."**

**In the discussion we note "The appearance of active CES in late spermatogonia indicates that dosage compensation (or a transcriptional trend with similar effects) is active in these cells and equalized X-autosome levels may not be solely due to retained transcripts produced from earlier cell stages" (lines 283-286).**

10. line 196: "close to an MSL CES" - MSL and CES sites are not always the same. I think you only need "CES" here. Regarding the effect of DCC distance on X expression in somatic or germline cells, a relevant reference is Belyi et al. (Genome Biol Evol. 2020 Dec 6;12(12):2391-2402).

**Response: This now reads "Close to a CES".**

11. line 337" "MSL CE sites" - see above comment. Should be CES?

**Response: We have changed this as requested.**

12. Discussion: As mentioned above, one of the most striking findings of the study is that there is "excess DC" in GSC/early spermatogonia. The authors should provide further discussion of this. Is the effect

chromosome-wide? Could it be a result of gene-specific regulation and, thus, not a form of dosage compensation?

**Response: Please see our response to the first point of the editors. We have added more discussion of this in lines 270-286.**

13. line 374: "Witt et. al 2019." - put parentheses around the year. The period should be after "al."

**Response: This has been fixed.**

14. line 344, Discussion of MSCI: was there a relationship between the expression level of genes and the X:autosome ratio? Previous work suggests that if one considers only genes with very high expression the X:autosome is reduced more than if genes with lower expression are included (see Argyridou et al. (Genes (Basel). 2018 May 4;9(5):242.)

**Response: Our X:autosome ratio compares median counts for all X chromosome genes and all autosomal genes, whether or not they are highly or lowly expressed, and is not gene-specific. Of note, in Figure 2, GSC/early spermatogonia and late spermatids have similar global levels of transcription, but the X:autosome ratios are very different between these two cell types. We added the suggested citation (Ref 34) in the discussion and noted that the apparent rise in X:autosome ratio between early and late spermatids could be a technical artifact due to globally lowered gene expression.**

**Response to reviewer 3:**

"…The concept of DC is important. Authors revealed several lines of evidence supporting the presence of DC in Drosophila testis and potentially novel mechanistic insights on the machinery of DC (likely non-canonical). Given these two lines of consideration, I think that this manuscript could be publishable. However, the current version has a room to be significantly improved."

**Response: We thank the reviewer for their positive feedback and critical evaluation. The comment on X-linked testis-specific genes is insightful. We actually performed an analysis after removing testis-biased/specific genes in the previous version (S8, S10, S11 figures), and now we revised in the text to make it clearer.**

Major concerns:

1. The strong expression of X-linked testis-specific genes have been interpreted as overcompensation. However, the consequence of DC is to balance the expressional output between X and autosome. So, for these testis-specific genes, DC seems not necessary. This is why testis-specific genes have been generally

excluded in the analysis of expression ratio between X and autosome (X:A ratio, e.g. Pubmed ID: 28132849).

**Response: Thank you for your comment. We understand that testis-specific genes are not directly relevant in DCC. In the S8, S10, and S11 figures, when we exclude testis-specific and testis-biased genes from our analysis, we still see evidence of pre-meiotic dosage compensation. Thus, our results still hold. The authors cited mentioned that "These genes are silent in the soma, and thus their inclusion can artificially lower estimations of the somatic X:A ratio". When we removed these testis-specific genes, X:A ratios did not change in our somatic cells. This is probably because our analysis only includes genes with detectable expression in a cell type, so our results are not susceptible to artificially lowered X:A ratios from silent genes in somatic cells.**

**One thing to note is that, in *Drosophila* testis-specific genes are thought to be depleted on the X chromosome and are enriched in autosome. Thus, it is interesting to think why X-linked testis-specific genes are extremely enriched in the earliest germ cells, but no other cells. In S8 figure, we show that after removing testis biased genes, we still see that, in GSC/early spermatogonia, genes near CES have a much higher expression than those who are not near CES sites. In S11 figure, we show that after removing testis-biased genes, counts from X genes in GSC/Early spermatogonia. are still higher than those from A (removing testis-specific genes showed a marginally insignificant result, p. adj=0.059). Together the results suggest that the high X is linked to a compensation-related mechanism and not sex-biased genes. We thus think the overcompensation is still a proper word. However, we are open to suggestions. We'd gladly change the word if the reviewers have a better suggestion.**

**On the other hand, we appreciate the comments from the reviewer, and agree that we should tone down the excess dosage compensation results. Please see the above response to editor (comment 1), which explained the changes we have made during revision.**

2. The writing or figure design is problematic.
a) Fig2/Table1 and Fig3/Table2 have been designed in the same way where X and autosome have been shown side by side, which is followed by statistics in Tables. I wonder why Fig1 (UMI distribution) is not designed in the same way.

**Response: Total X and autosomal UMI ratios are a poor proxy for dosage compensation because they can be influenced by a small number of outlier genes. This figure is just meant to compare RNA levels between cell types, and X:autosome ratios can be better inferred by the methods used for the later figures which control both for gene numbers and the presence of outliers. We feel like**

**this result does not need to be emphasized the same way as Figure 2 and 3, thus, we put the corresponded table as S1 table (corresponds to Figure 1C-D).**

b) To highlight the key discovery of this work, I suggest authors to add a figure in Discussion to summarize the dynamic picture of DC, the underlying complexity of DC machinery (non-canonical) and how this work is in line with the previous related work (e.g. Mahadevaiah et al., 2020; Mahadevaraju et al., 2020). In this aspect, authors mentioned "msl-3 might instead be facilitating entry into meiosis…the lack of germline enrichment of any other DCC genes". Does this mean that authors believed the machinery underlying pre-meiotic DC is entirely different from the canonical one?

**Response: The absence of expression for most DCC components suggests that premeiotic DCC is likely not mediated by the canonical DCC. Based on our observation and the pioneer work from Rastelli & Kuroda (1998), MSL-3 is likely to play other roles than dosage compensation in the germline, because it cannot perform DC by itself. Our results combined with Mahadevaraju et al., (2020) suggest that the non-canonical dosage compensation can be found from both larvae and adult testis, further support that this observation is unlikely to be spurious. There is a possibility that germline DC is mediated by an unknown complex – either partly or completely different from DCC - from the same sequence elements as canonical DC, since the entry site analysis suggest that dosage is correlated to the canonical entry sites. There are still many unanswered questions about this potential noncanonical pathway, and we think a model should be proposed after the appropriate genetics work has been done to clarify the potential components of such a pathway. We appreciate the reviewer's encouragement. We edited the discussion to reflect this idea.**

3. Authors mentioned Clamp as one essential protein of DCC. However, in fig. 4, this gene is not covered. Could authors explain why? In addition, "no DCC genes were enriched in germ cells except msl-3 and Clamp, which were enriched in GSC/early spermatogonia (Figure 4A)." Again, Clamp is not shown in Fig. 4A.

**Response: Thank you for pointing out the omission. We added Clamp to figure 4A.**

Minor concerns:

1. "others to suppress it in females". I guessed that authors referred to human system. However, for human, an additional mechanism acts to upregulate single active X chromosome to balance X and autosomal transcription. So, writing should be revised here to be more specific.

**Response: Thank you for the comment. Here we hope to convey that many species use different mechanisms for dosage compensation without going into details. We changed this sentence to read "others to randomly inactivate one X or suppress both X chromosomes in females". We also added two reviews Sangrithi & Turner 2018 and Samata & Akhtar 2018, in case the readers are interested in further readings of this topic.**
